# Frequency Specificity of Regional Homogeneity in the Resting-State Human Brain

**DOI:** 10.1371/journal.pone.0086818

**Published:** 2014-01-23

**Authors:** Xiaopeng Song, Yi Zhang, Yijun Liu

**Affiliations:** 1 Department of Biomedical Engineering, College of Engineering, Peking University, Beijing, China; 2 School of Life Science and Technology, Xidian University, Xi’an, Shanxi, China; National Taiwan University, Taiwan

## Abstract

Resting state-fMRI studies have found that the inter-areal correlations in cortical networks concentrate within ultra-low frequencies (0.01–0.04 Hz) while long-distance connections within subcortical networks distribute over a wider frequency range (0.01–0.14 Hz). However, the frequency characteristics of regional homogeneity (ReHo) in different areas are still unclear. To examine the ReHo properties in different frequency bands, a data-driven method, Empirical Mode Decomposition (EMD), was adopted to decompose the time series of each voxel into several components with distinct frequency bands. ReHo values in each of the components were then calculated. Our results showed that ReHo in cortical areas were higher and more frequency-dependent than those in the subcortical regions. BOLD oscillations of 0.02–0.04 Hz mainly contributed to the cortical ReHo, whereas the ReHo in limbic areas involved a wider frequency range and were dominated by higher-frequency BOLD oscillations (>0.08 Hz). The frequency characteristics of ReHo are distinct between different parts of the striatum, with the frequency band of 0.04–0.1 Hz contributing the most to ReHo in caudate nucleus, and oscillations lower than 0.02 Hz contributing more to ReHo in putamen. The distinct frequency-specific ReHo properties of different brain areas may arise from the assorted cytoarchitecture or synaptic types in these areas. Our work may advance the understanding of the neural-physiological basis of local BOLD activities and the functional specificity of different brain regions.

## Introduction

Functional segregation and integration are the two guiding principles that shaped brain mapping at its inception [1], [3] These two principles reflect the distributed and integrated nature of neuronal processing [2]. Neuroimaging has established functional segregation (the segregated or modular deployment of functional specialization within brain regions) as a fundament of brain organization, and has established functional integration (the co-activation or driving-driven relationship of spatially remote brain regions) as a possible mechanism for parallel processing [1] and brain resource integration [2], [3]. Functional integration is more relevant to the global properties of the brain, whereas functional segregation is dominated by the local properties of different brain regions. In blood-oxygenation-level-dependent fMRI (BOLD-fMRI) studies, the synchronization or causal relationship between distant brain regions are usually measured with functional connectivity (FC) and effective connectivity (EC) [2], [3], while the local features of brain activity can be characterized by the Regional Homogeneity (ReHo) [4] measurement. Mathematically, FC quantifies the synchronization of the BOLD time courses of distant voxels or brain regions by calculating Pearson correlation or partial correlation coefficients, while ReHo is a voxel-wise measure of the synchronization of the time courses of a certain voxel and its adjacent neighboring voxels.

Both FC and ReHo have been widely applied to resting-state fMRI (RS-fMRI) studies. Since the seminal study by Biswal et al. [5], numerous fMRI studies have found that during rest, the BOLD signals of some nodes in different sites of the brain oscillate in a synchronous way. These nodes constitute the so-called resting-state networks (RSNs). A number of RSNs that are relevant to critical functions such as vision, motor planning, memory and attention directing [6], [7] have been delineated. Of particular interest is a unique network dubbed the default mode network (DMN): a network thought to be involved in “mind wandering” [8], sleep [9], self-referential thoughts [10], and to maintain the brain in an idle and “ready” or “default” mode [11]. These findings suggest that spontaneous resting-state oscillations are robust representations of the state of human brain in the absence of goal-directed neuronal activation and external input.

One critical step toward understanding the neural-physiological basis of resting-state BOLD activities is to clarify the spectral characteristics of FC and ReHo. Results of electrophysiological studies show that gamma-band (30–100 Hz) neural activity contributes to local BOLD signals [12], [13], whereas low-frequency rhythms (<20 Hz), not gamma activity, predominantly contributed to inter-areal BOLD correlations [14]. These observations implied that the neural origins of FC and ReHo may not be exactly the same and the frequency characteristics of inter-areal and local BOLD activities may be distinct [15]. Some RS-fMRI studies have investigated the frequency-specific characteristics of FC [15] and found that the inter-areal correlations in cortical networks concentrated within ultra-low frequencies, (0.01–0.04 Hz) while long-distance connections within subcortical networks distributed over a wider frequency range (0.01–0.14 Hz); however, very few studies have explored the frequency-specific characteristics of ReHo in different brain regions.

Previous studies have attempted to delineate the spectral characteristics of the BOLD signal by dividing the whole bandwidth into arbitrarily determined smaller frequency bins and then estimating the Fourier power spectra or functional connectivity in each of the bins [16], [17]. However, in these studies, the whole frequency band was divided into small bins arbitrarily and without any sound justification. Moreover, analyzing BOLD time series solely with Fourier transform may be overly simplistic due to the inherent assumptions of linearity and stationarity in a Fourier analysis—two assumptions that have not been validated in the context of BOLD activities [18]. On the other hand, the Empirical Mode Decomposition (EMD) [19] can avoid the disadvantages of the above methods, it automatically isolates the underlying processes of BOLD activities in a data-driven manner and divides the whole frequency band into adaptively determined sub bands without any assumption of linearity, stationarity, or recourse to any rigid priori chosen band-pass filter. EMD decomposes a time series into several components called intrinsic mode functions (IMFs). Each IMF occupies a unique frequency range: the first IMF occupies the highest frequencies, and the last occupies the lowest, with the other IMFs in between.

With the purpose of investigating the frequency-specific characteristics of ReHo, in the current study, EMD was applied in a voxel-wise fashion to adaptively decompose the time series of each voxel into several IMFs. We then calculated ReHo in each IMF of each voxel and classified the voxels into different clusters based on these ReHo values. The frequency-specificity properties of ReHo in these clusters were analyzed. We attempted to answer the questions: through what frequencies are neighboring voxels correlated and how do frequency-specificity properties of ReHo vary from region to region in the resting-state human brain to reflect functional specificity?

## Methods

### 1 MRI Data Acquisition

The current research was approved by the Institutional Review Board of Peking University. FMRI data were acquired from the open source website (http://fcon_1000.projects.nitrc.org/fcpClassic/FcpTable.html) provided by ‘1000 Functional Connectomes’ Project. The resting state fMRI scans of all 198 healthy subjects (ages from 18–26 years old, 122 females) included in a dataset provided by Beijing Normal University were analyzed in the current study. No subject had a history of neurological, psychiatric, or medical conditions. Written, informed consent was obtained prior to scanning of all subjects in accordance with Institutional Review Board guidelines of Beijing Normal University and in compliance with the Declaration of Helsinki.

Imaging was performed using a 3.0-Tesla scanner (Siemens TRIO TIM, Munich, Germany). The participants were instructed to rest with their eyes closed, keep their heads still, and not to fall asleep. A gradient echo T2*-weighted EPI sequence was used for acquiring resting state functional images with the following parameters: TR  =  2000 ms, TE  =  30 ms, flip angle  =  80 degrees; matrix size  =  64×64, FOV  =  240×240 mm^2^, giving an in-plane resolution of 3.75 mm×3.75 mm, 51 axial slices (3.5 mm thickness with a gap of 1.2 mm). The scan for RS-fMRI lasted for 450 seconds, containing 225 brain volumes.

### 2 Image Preprocessing

Images were analyzed by using the following procedure with SPM8 (http://www.fil.ion.ucl.ac.uk/spm). The first 5 time points were removed to eliminate non-equilibrium effects of magnetization. The remaining 220 volumes of functional BOLD images were corrected for slice timing effects, motion corrected and spatially normalized to the Montreal Neurological Institute (MNI) template using the standard EPI template, resulted in functional image series of 61×73×61 voxels (voxel size of 3 mm×3 mm×3 mm). No translation or rotation parameters in any given data set exceeded ±2 mm or ± 2 degree. These images were not spatially smoothed as previous studies suggested [4]. Linear trend was regressed out from each voxel’s time course, to remove signal drifts that arise from scanner instability or other causes. The time course of each voxel was then normalized by subtracting its own temporal mean and dividing by its own temporal standard deviation.

### 3 Empirical mode decomposition

The EMD method [19] decomposes the original signal into a finite set of intrinsic oscillatory components, termed IMFs. Mathematically, for a real-valued BOLD signal, the standard EMD finds a set of *K* IMFs {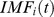}, *i* = 1 to *K*, and a monotonic residue signal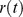, so that
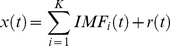
(1)


To ensure that the time frequency spectra yields meaningful frequency estimates (e.g. no negative frequencies), IMFs{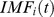} are defined so as to have symmetric upper and lower envelopes, with the number of zero crossings and the number of extrema differing at most by one. To extract IMFs using EMD, an iterative method known as sifting algorithm is used; for illustration, a sifting procedure for obtaining the first IMF (IMF1) from the signal is outlined in the algorithm below.

The standard EMD algorithm:

1) Find the locations of all the extrema of;

2) Interpolate between all the minima (resp. maxima) to obtain the lower (resp. upper) signal envelope, 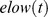 (resp.);

3) Compute the local mean time course;

4) Obtain the “oscillatory mode” from 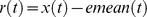;

5) If 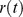 obeys the stopping criteria, 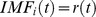 becomes an IMF, otherwise set 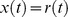 and repeat the process from Step 1.

To obtain remaining IMFs, the same procedure is applied iteratively to the residual 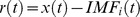
until we are left with the monotonic signal. The standard stopping criterion terminates the sifting process only after the IMF condition is met for *S*consecutive times ( *S* is normally taken to be 2 or 3), here S = 3.

**Figure 1:**
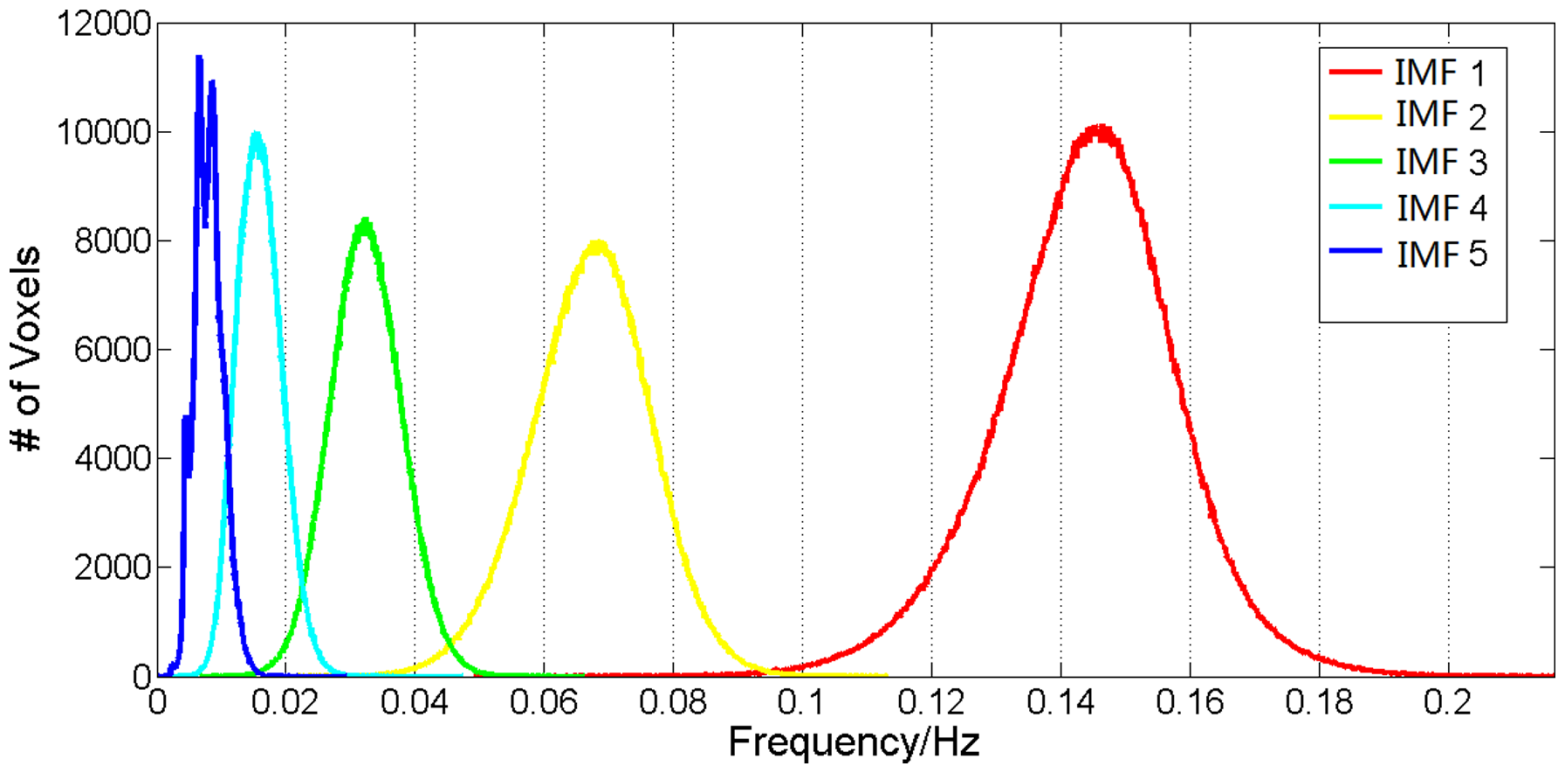
Frequency properties of IMFs. The histograms of HWF of IMF1 to IMF5 (color-coded by red, yellow, green, cyan and blue respectively) were determined from all the voxels in the whole brains across all the 198 subjects. Heights of the histograms represent the number of voxels whose HWF equals that frequency on the horizontal axis.

The Hilbert weighted frequency (HWF) [20] of each IMF was determined using instantaneous information about amplitude and phase to reflect the mean oscillation frequency of the IMF. HWF is comparable to the traditional Fourier frequency and is measured by units of Hertz. The HWF reflects the mean frequency of an IMF by summarizing its spectral characteristics. For example, if the HWF of an IMF time course equals 0.15 Hz, it indicates that more energy is distributed around 0.15 Hz and the Fourier power spectrum of this IMF peaks around 0.15 Hz. The discussion of how to calculate HWF is beyond the scope of this work and the reader is referred to the literature for a more detailed description of the calculation [20]. For most voxels, the decomposition of the time course yielded only four to five IMFs and the frequency range of the first five IMFs covers 0–0.25 Hz. Consequently, only the first five IMFs of each voxel were considered in this study, denoted as IMF1 to IMF5, and we calculated the HWF of IMF1 to IMF5 for each voxel to get the histograms of HWF distribution of IMF1 to IMF5 for the voxels in the whole brain (**Figure** **1**). We intended to test whether the frequency of the same IMF (IMF_i_, i = 1, 2, 3, 4 or 5) for all the voxels across the brain and subjects were roughly similar and were generally higher than the frequency of the next IMF (IMF_i+1_).

### 4 Regional homogeneity

After finishing the aforementioned preprocessing steps, the original time course of each voxel was decomposed into several IMF time courses, and the first five IMFs were ready for ReHo analyses. Data were analyzed using the resting-state fMRI Data Analysis Toolkit (http://www.restfmri.net/forum/index.php). ReHo analysis was performed for each IMF of each intracranial voxel by calculating the Kendall’s coefficient of concordance (KCC) of the IMF of the voxel with those of its nearest neighbors (26 voxels) in a voxel-wise fashion:
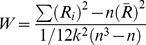



Where 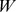 ranges from 0 to 1; 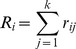 where 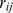 is the rank of the *i* ^th^ time point in the *j* ^th^ voxel; 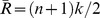 is the mean of the 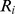, 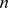 is the number of time points of each IMF time series (here n  = 230), and k is the number of time series within the measured cube of voxels (here k  = 27, the central voxel plus its 26 neighbors). Thus, we obtained a ReHo value for each of the first five IMFs of the voxel, i.e. five ReHo values for each voxel or five ReHo maps for each subject. We also chose k = 7 and 19 as indicated in the reference [4] to see whether the neighborhood size influences the results.

To compute statistical significance across subjects and determine the regions in which the ReHo differs significantly among the five frequency bands (IMFs), a voxel-wise one-way ANOVA was performed on the ReHo maps across different frequency bands. FDR corrections for multiple comparisons were executed with a voxel-wise threshold of *P* < 10^−5^ and a minimum cluster size of 200 voxels, yielding an overall false positive *Q* < 0.001.

### 5 k-means clustering analysis based on the frequency characteristics of ReHo

In order to investigate whether different functional regions could be distinguished based on the frequency characteristics of ReHo; a k-means clustering method was applied to the data of each subject to classify voxels into different classes [21]. The Five ReHo values for each voxel were used as features when performing k-means clustering analysis, assigning each voxel a frequency-specific ReHo signature. Voxels fall naturally into clusters, where the frequency-specific ReHo characteristics of voxels in the same cluster are similar, and the frequency-specific ReHo characteristics of voxels in different clusters are distinct. For instance, two voxels have their highest ReHo in IMF3, and exhibit similar lower ReHo values across other IMFs, will fall into the same cluster since both of them are synchronized with their own surrounding voxels mainly through IMF3 waves. If one voxel has overall higher ReHo across all the IMFs than another voxel, they will fall into different clusters. We intended to investigate whether or not two voxels that are anatomically close to each other (e.g. one from V1 and the other from V2) or functionally belong to one canonical RSN (e.g. one from posterior cingulate cortex and the other from inferior parietal lobule) will also exhibit similar ReHo values across IMFs.

**Figure 2:**
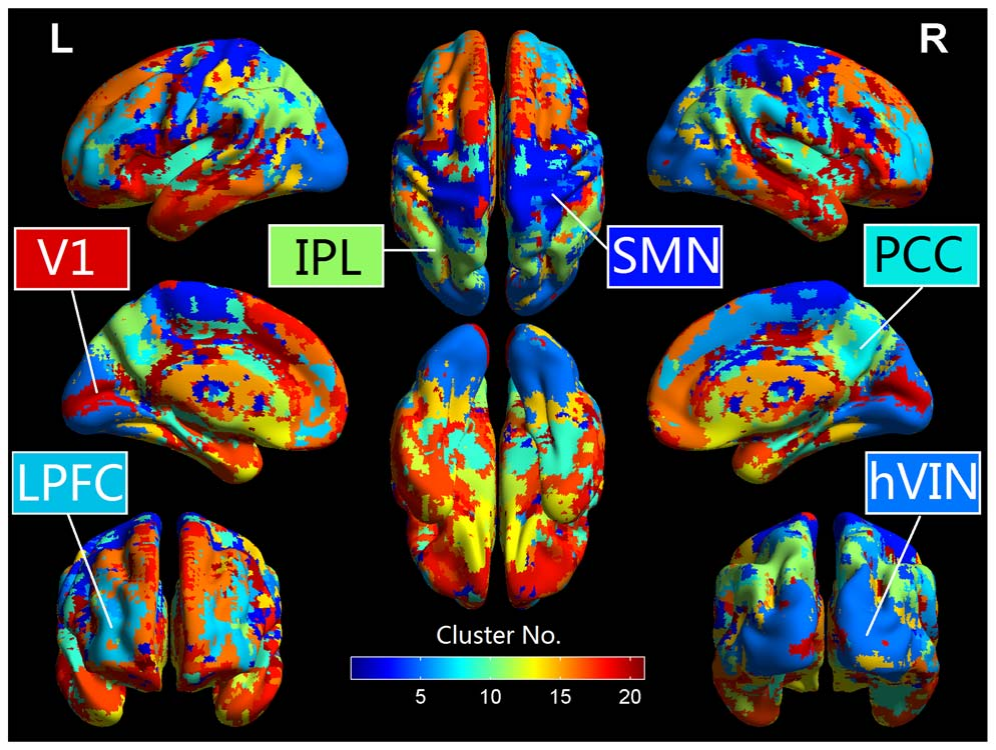
Result of k-means clustering. Voxels with similar frequency-specific ReHo characteristics were automatically classified into the same cluster. Different clusters are coded with different colors. Certain canonical cortical regions (networks) are identified and labeled include: posterior cingulate cortex (PCC)/precuneus, bilateral inferior parietal lobule (IPL), lateral prefrontal cortex (LPFC), primary visual area (V1), higher-order visual network (hVIN).

**Figure 3:**
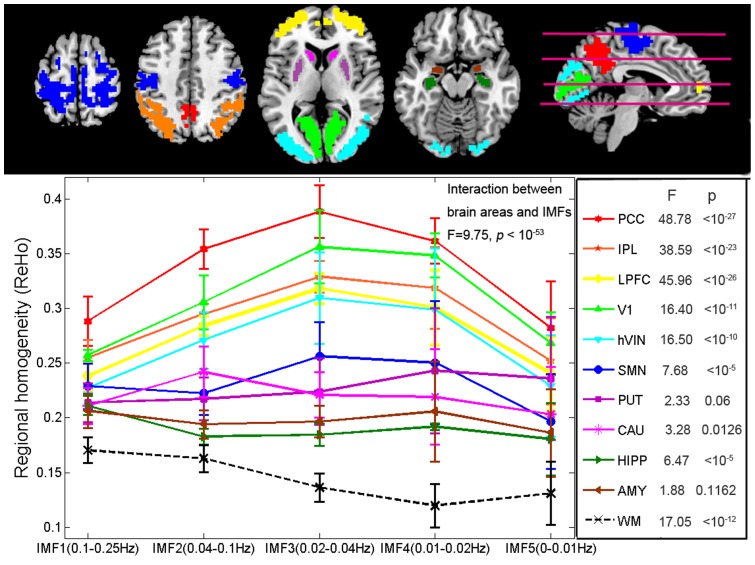
Frequency-specific ReHo in different brain regions. Upper panel: The conjunction masks created from the clustering results. Lower left panel: The ReHo values were averaged across all the voxels within each of the brain regions/networks for each subject. Group mean and standard errors (SE) of ReHo for each IMF were then determined across the 198 subjects. Lower right panel: F-scores and corresponding P values from the balanced one-way ANOVA performed for each of the brain regions/networks. Abbreviations: posterior cingulate cortex (PCC)/precuneus, bilateral inferior parietal lobule (IPL), lateral prefrontal cortex (LPFC), primary visual area (V1), higher-order visual network (hVIN), sensory motor network (SMN), putamen (PUT), caudate (CAU), hippocampus (HIPP), amygdala (AMY) and white matter (WM).

We set the range of clusters, k, from 10 to 60 in order to find the appropriate number of clusters [21]. There was a sudden decrease of the within-cluster variations beginning with k  = 21 and extending to k  =  27. This decrease indicated an appropriate range for the choice of cluster numbers. Since the choice of k within this appropriate range would not affect the results strongly, we chose k = 21 for all the subjects. To illustrate a representative clustering result (**Figure 2**), we concatenated the ReHo maps of the 198 subjects (a total of 5*198 = 990 ReHo maps) and performed k-means clustering analysis. In this case, a voxel in a specific location of the brain was classified based on 990 feature variables from all the subjects,

To investigate the frequency characteristics of ReHo in different brain regions, some representative brain areas/networks were identified from the resulting clusters of each subject by using a template-matching procedure. For each cluster, we counted the number of voxels falling within a certain template minus the number of voxels outside the template and selected the cluster in which this difference was the greatest. Standard atlas of Brodmann areas (BAs) and Automated Anatomical Labeling (AAL) regions were used as templates to select the clusters corresponding to the following brain areas/networks: posterior cingulate cortex (PCC)/precuneus(AAL67,68,35,36), bilateral inferior parietal lobule (IPL/AAL61,62), lateral prefrontal cortex (LPFC/AAL7,8), primary visual area (V1/BA17), higher-order visual network (hVIN/BA18-19), and sensory motor network (SMN/BA1-5). The following four subcortical areas were also identified: bilateral putamen (PUT/AAL73,74), caudate nucleus (CUA/AAL71,72), hippocampus (HIPP/AAL37,38) and amygdala (AMY/AAL41,42). Symmetric bilateral AAL templates were merged as one template when used to select corresponding clusters since all these clusters were bilateral. BA18-19 and BA1-5 were merged as one template when used to select hVIN and SMN respectively since clusters corresponding to these two networks usually covers more than one BA. After the clusters corresponding to a certain brain area were identified for all the subjects, the intersection of these clusters was calculated to create a common mask (i.e., conjunction mask) for all the subjects. We removed the outer layers of the masks and only used the inner parts of the masks to ensure that each remaining voxel in the modified masks is surrounded by 26 voxels within the same functional region (**Figure 3**). The ReHo values were averaged across all the voxels within each of these masks for each subject. Group mean and standard errors (SE) of ReHo for each IMF were then determined across the 198 subjects. For comparing the group mean of ReHo in the five frequency bands (IMFs), a balanced one-way ANOVA was performed for each of the selected brain areas (i.e. identified clusters). For testing the effects of two factors, brain areas and frequency bands, on ReHo, a balanced two-way ANOVA was performed to examine whether there was interaction between these two factors. P<0.05 in ANOVA was considered as a threshold of significant difference.

## Results

We first investigated the frequency properties of each IMF component. The histograms of HWF distributions for the first five IMFs of the voxels in the whole brain across all the subjects were shown in **Figure 1**. Each of the five histograms is a statistic of the whole-brain voxels. Since the frequency content of different voxels at different sites of the brain (and subjects) are roughly similar, the same IMF (IMFj, j = 1, 2, 3, 4 or 5) from any voxel will roughly fall into the same frequency band. Hence, the Hilbert weighted frequencies of IMF1 of all the voxels in the brain range from 0.095 to 0.22 Hz and are generally higher than the frequencies of IMF2; this is the same with the other IMFs. As shown in Figure 1, each IMF occupies a unique frequency band with very slight overlap: the first IMF (IMF1) occupies the highest frequencies (0.095–0.22 Hz); the frequencies of IMF2 of all the voxels range from 0.04 to 0.1 Hz; IMF3 from 0.02 to 0.05 Hz; IMF4 from 0.01 to 0.03 Hz; and IMF5 occupies the lowest frequency band 0 to 0.015 Hz. These frequency properties of IMFs make it possible for us to compare the ReHo of the same IMF across different brain areas. These results suggest that EMD works well in adaptively decomposing the original time series into different intrinsic oscillatory modes that fall into distinctive frequency bands and is a promising method for non-stationary and non-linear neurological signal processing.

The k-means clustering results (**Figure 2**) showed that the spatial distribution of some clusters are consistent with cortical regions (brain networks) that are associated with certain brain functions, and have been well-studied in previous studies [6], [7]. It is noteworthy that PCC, IPL and LPFC, which have always been found to be pivotal parts of DMN in functional connectivity studies, were classified into different clusters based on their distinct frequency-specific regional properties in all the subjects. The mask of LPFC (**Figure 3**) obtained from the clustering results also includes part of medial prefrontal cortex (MPFC). The primary visual cortex (V1/ BA 17), which has been found to be correlated with other visual areas to constitute the VIN, was also segregated from the other higher-order visual areas (V2, V3 and V4/BA 18, 19) in the study.

The clusters corresponding to the aforementioned cortical areas (networks) together with four subcortical areas, bilateral PUT, CAU, HIPP and AMY, were chosen as ROIs to investigate the frequency-specific characteristics of ReHo in these areas. We also analyzed ReHo in the cluster corresponding to white matter to serve as a reference. The spatial patterns of all these clusters are bilateral, and are formed from anatomically disjoint regions from both hemispheres. The results (**Figure 3**) indicated that cortical areas showed consistent higher ReHo than subcortical regions across the whole frequency band, white matter has the lowest ReHo in all the five frequency bands. In line with the results of previous studies that, during rest, ReHo was higher in the major regions of DMN (PCC, IPLs, MPFC and LPFC) [22], we also found that ReHo in PCC, IPLs and LPFC are generally higher than other brain areas across the whole frequency band. Furthermore, even within the same network, ReHo varies significantly from region to region: In DMN, the PCC showed higher ReHo than IPL and LPFC; in the visual network (VIN), the V1 showed higher ReHo than hVIN.

For regions within DMN (PCC, IPL and LPFC) and VIN (V1 and higher-order visual areas), the ReHo values in IMF3 (0.02–0.04 Hz) were the highest among the five IMFs, followed by IMF4 (0.01–0.02 Hz). For SMN, ReHo values in IMF1 (0.08–0.25 Hz) were also very high besides that in IMF3 and IMF4. In putamen, IMF4 and IMF5 (0–0.01 Hz) contribute most to ReHo, while in CAU, IMF2 contributes more to ReHo than the other IMFs. For HIPP and AMY, IMF1 had the highest ReHo values, followed by IMF4, but ReHo in the other IMFs were not significantly different from each other. We also chose k = 7 (Figure SI1) and 19 (Figure SI2) as the neighborhood size in ReHo calculation and have found similar results. The results of one-way ANOVA and multiple comparisons (Figure 3, right panel) showed that ReHo in neocortical networks (DMN, VIN and SMN) is more frequency-dependent and is dominated by IMF3 and IMF4 (F-value>7.68, *P* < 10^−5^). ReHo in CAU is dominated by IMF2 (F-value = 3.28, *P*  = 0.0126), while ReHo in HIPP is dominated by IMF1 (F-value = 6.47, *P* < 10^−5^) with no significant difference of ReHo among the other four IMFs. There’s no significant difference of ReHo between the five frequency bands in AMY (F-value  = 1.88, *P*  =  0.1162) and PUT (F-value  = 2.33, *P*  =  0.06). The results of two-way ANOVA showed that ReHo varied significantly across different locations (brain areas as the main factor, F = 258.66, *P* < 10^−200^) and different frequency bands (IMF as the main factor, F = 82.63, *P* < 10^−65^). There is also significant interactions between these two factors, location and frequency (F = 9.75, *P* < 10^−53^). This interaction implies that the frequency properties of ReHo are different from region to region: the five IMFs contribute differently to the ReHo in some brain areas but equally in some other areas.

**Figure 4:**
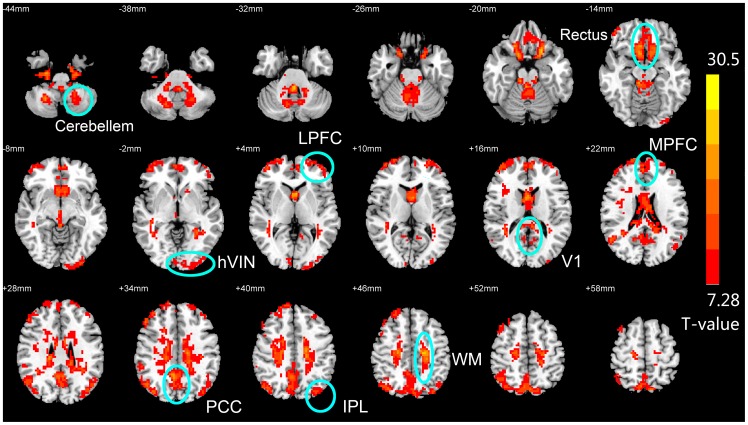
F-map (Q<0.001, FDR corrected) representing brain areas whose ReHo are significantly frequency-dependent. Key brain areas survived the FDR correction are marked with a circle and the names (abbreviations) of these areas are indicated beside the circle. Abbreviations: posterior cingulate cortex (PCC)/precuneus, bilateral inferior parietal lobule (IPL), lateral prefrontal cortex (LPFC), primary visual area (V1), higher-order visual network (hVIN), sensory motor network (SMN), and white matter (WM).

The frequency dependence of ReHo in different brain regions can also be demonstrated in F-score maps from the voxel-wise ANOVA (Q value < 0.001, with FDR correction, **Figure 4**), with frequency (IMF) as the main factor. A voxel with high F-value indicated that ReHo of this voxel is significantly different across the five frequency bands. Results showed that ReHo in PCC, MPFC, bilateral IPLs, bilateral LPFC, V1, higher-order visual cortex, rectus, cerebellum and white matter are significantly frequency-dependent, and distinct IMFs contribute differently to the ReHo in these areas. White matter came up as one of the larger regions exhibiting frequency-dependent ReHo because the synchronization of neighboring voxels was mainly through IMF1 (>0.1 Hz) and other IMFs contributed little to ReHo in WM. However, even in IMF1 the absolute value of ReHo in WM is very low and significantly lower than that in the other brain regions, which means that neighboring voxels in WM are barely synchronized with each other. The slight synchronization of neighboring voxels in WM through IMF1 might be resulted from physiological noises.

## Discussion

Both fMRI and electrophysiological studies have identified that several brain rhythms of independent frequency bands could temporally coexist in the same or different brain structures and may interact with each other [13], [18]. These rhythms may be generated by distinct oscillators with specific properties and physiological functions [13], [18]. EMD proved to be a useful technique in isolating these underlying coexisting rhythms in different frequency bands [18]. The frequency interval covered by each of the IMFs is distinctive, and is consistent with the sub-frequency bands defined by Buzsaki et al.[23]. Frequency bands division defined by Buzsaki was derived from the scale-free dynamics of brain activities [24], [25]; our IMF1 corresponded to slow-3 waves defined by Buzsaki et al., IMF2 and IMF3 corresponded to slow-4, and IMF4 corresponded to slow-5 waves. By using EMD, we provided conclusively the justifiable division of the full frequency band of BOLD signal.

The k-means clustering results indicated that the frequency characteristics of ReHo varied from region to region, and could be used to demarcate the anatomically or functionally segregated brain regions. In previous FC studies, PCC, IPLs and LPFC/medial prefrontal cortex (MPFC) were usually found to correlate with each other to form DMN [11], and V1 was also integrated with the anatomically adjacent higher order visual cortices to form VIN [6], [7]. However, in the current study, PCC, IPLs, and LPFC/MPFC were found to be separated with each other according to the clustering results. The V1 and hVIN also fell into different clusters. These differences between the FC networks and the spatial patterns of ReHo-based clusters suggested different neural physiological implications for FC and ReHo: FC reveals common activities and temporal similarities of distant brain regions and reflects functional integration, whereas ReHo emphasizes the aggregation of neighboring neuronal assemblies and reflects regional functional specificity [4]. Although PCC, IPLs and LPFC/MPFC are important components of the same network, DMN, each of these nodes has its own specific regional properties, so are the different parts of VIN. In fact, these nodes or brain areas are anatomically equivalent to different Brodmann areas (BAs), and each of the BAs has distinct cytoarchitectonics, or structure and organization of cells [26]. We conjectured that the distinct frequency-specific ReHo properties for different brain areas might arise from the assorted cytoarchitecture of these areas [17].

We found that brain areas with simple functions, such as V1 and PCC, show higher ReHo than areas with complex functions, such as higher-order visual areas and LPFC. Cortical areas showed also higher ReHo than subcortical areas. The overall ReHo in the motor-related system (including SMN, PUT and CAU) is lower than that in the other cortical areas but higher than that in the limbic system (HIPP and AMY). We speculated that brain areas with more complex or multiple functions would show generally lower ReHo during rest because the underlying neural activities are more complex [17], [27]. Multiple ongoing neural processes may co-exist in the same area within a small volume and be maintained by brain waves of different frequencies [18], [23], these processes may be reflected by the ReHo in different frequency bands. ReHo in specific frequency bands may further be modulated by different tasks [28], stimuli or pathophysiological states [29]. For example, the SMN mask comprises precentral, postcentral gyrus and supplementary motor areas, these areas are responsible for sensory information processing, motor planning, initiation and execution. Since most of these functions or underlying processes are not activated during rest, they may co-exist in the resting-state SMN and interact with each other, resulting in an overall lower ReHo than in the other cortical areas. One recent study indicated that, when performing a certain task, some of the co-existing processes might dominate the local activities and the ReHo would be up regulated in related frequency bands and down regulated in some other bands [28]. When the task was changed, some other processes might take over from the current ones, and the frequency characteristics of ReHo would be further altered [28]. This hypothesis is also consistent with the results of other fMRI studies: compared with the resting-state, BOLD trajectories become more constrained [30] and the power distribution across frequencies becomes narrower during task-state [31]. Further task-based fMRI studies are needed to confirm this supposition.

A number of RS-fMRI studies on the frequency-specific characteristics of FC have shown that functional connectivity in cortical networks were more frequency-dependent and concentrated within 0.01–0.04 Hz, while correlations within limbic networks were less frequency-dependent and distributed over a wider frequency range (0.01–0.14 Hz) [15]. Similar to these FC findings, our results showed that the frequency-specific features of ReHo for cortical and subcortical areas were also distinct: while IMF3 (0.02–0.04 Hz) and IMF4 (0.01–0.02 Hz) waves contributed most to the cortical ReHo, IMF1 waves (>0.08 Hz) made a significant contribution to the limbic ReHo. While the ReHo distribution patterns are similar between HIPP and AMY, the frequency characteristics of ReHo are distinct between different parts of the striatum: IMF2 waves (0.04–0.1 Hz) contributed the most to ReHo in CAU, whereas IMF4 and IMF5 waves (0–0.02 Hz) contributed more to ReHo in putamen. Although the overall ReHo of SMN is similar to that of the striatum, the ReHo distribution patterns across different IMFs in SMN are still more similar to that of the other cortical areas, with IMF3 and IMF4 contributing the most to ReHo (**Figure 3**). These results are in line with previous findings that the energy of low-frequency (<0.08 Hz) BOLD oscillations is higher in cortical areas, while the energy of high-frequency (>0.08 Hz) BOLD oscillations is higher in subcortical areas [16], [17]. ReHo within subcortical areas are more evenly distributed across the whole frequency band than those within cortical areas. These results indicated that subcortical areas recruit multiple frequencies to maintain local coordination, whereas in neocortical areas, different frequencies contributed very differently to ReHo, and ReHo in cortical areas were dominated by a narrower lower frequency band (0.01–0.04 Hz).

The distinct frequency characteristics of ReHo in different brain areas might result from the different cytoarchitecture and synaptic linkage types among them [27]. Mesulam [27] categorized the brain areas into three clusters based on cytoarchitectonic and functional grounds: unimodal, multimodal and transmodal areas. According to this classification, areas of neocortical networks, such as DMN, VIN and SMN, are mostly unimodal and multimodal areas, while limbic and paralimbic regions like HIPP and AMY are transmodal areas. Unimodal areas are the most cytoarchitecturally differentiated regions, encoding basic features of sensation or motor output and only exchange information with limited cortical regions; multimodal areas are more hierarchically organized than the unimodal areas [32]; transmodal areas transfer information from one brain area to another and are composed of neural cells with more diversities, complexities and heterogeneous connectivity [17], [27]. Recent studies have found that the BOLD spectral properties and FC are distinct among unimodal, multimodal and transmodal areas, and are related with the regional synaptic wiring [31]. We inferred that both the synaptic linkage types and the underlying neural activities were more diverse and heterogeneous in the brain areas for complex functions [27], and these diversities lead to a less homogeneity within these areas. The difference in ReHo properties between cortical (mainly unimodal and multimodal BAs) and subcortical areas (mainly transmodal areas) may hence arise from the difference in synaptic/functional/cytoarchitectonic complexity [17]. Further works combining BOLD-fMRI, electrophysiological data and brain structural data are needed to answer the question why different brain areas recruit brain waves in distinct frequency bands to maintain the coordination of local activities.

Electrophysiological studies have found that gamma-band (30–100 Hz) neural activity contribute to local BOLD signals [12], [13], and low-frequency neural oscillations (<20 Hz) not only contributed to inter-areal FC, but also influenced local processing by modulating gamma activity within individual areas [14], implying the important role of low-frequency brain rhythms in both local and inter-areal BOLD activities. Our BOLD-fMRI results showed that the frequency band of 0.02–0.04 Hz, which predominately contributed to cortical FC [15], also contributed a lot to cortical ReHo. These results indicated that local and inter-areal BOLD activities differ from each other, but at the same time, might share some similar neural basis, and the regional homogeneity might serve as the fundament of distant correlation: a brain area needs to build up local synchronization of neighboring neurons within itself before it interacts with other distant areas [23].

## Conclusion

By combining EMD with ReHo, we have intrinsically divided the whole band width of frequencies available with fMRI signal into several sub-bands, and studied the properties of ReHo in these frequency bands across different brain regions. Our results showed that cortical areas consistently possess higher ReHo than subcortical regions across the whole frequency band. While BOLD oscillations of 0.02–0.04 Hz mainly contributed to cortical ReHo, the ReHo in limbic areas involved a wider frequency range and were dominated by higher-frequency BOLD oscillations (>0.08 Hz). The frequency characteristics of ReHo are distinct between different parts of the striatum, with the frequency band of 0.04–0.1 Hz contributing the most to ReHo in CAU, and 0–0.02 Hz contributing more to ReHo in putamen. The distinct frequency-specific ReHo characteristics in different brain areas may reflect the assorted cytoarchitecture of these areas. To the best of our knowledge, this is the first study to clarify the frequency specificity of ReHo in different brain areas. We have provided a new method of analyzing local activity, which could reveal more information and might be more sensitive to different tasks [28] or pathophysiological states [29]. Our findings may advance the understanding of the neural-physiological basis of regional structural-functional specificity and its relationship with regional homogeneity.

## Supporting Information

Figure S1 **Frequency-specific ReHo in different brain regions, ReHo calculated with cluster size k = 7.**
(TIF)

Figure S2 **Frequency-specific ReHo in different brain regions, ReHo calculated with cluster size k = 19.**
(TIF)
