# Supplementary figures and images for: Frequency Specificity of Regional Homogeneity in the Resting-State Human Brain
Source: PLoS One. 2014 Jan 23;9(1):e86818. doi: 10.1371/journal.pone.0086818 (PMC3900644; doi:10.1371/journal.pone.0086818)

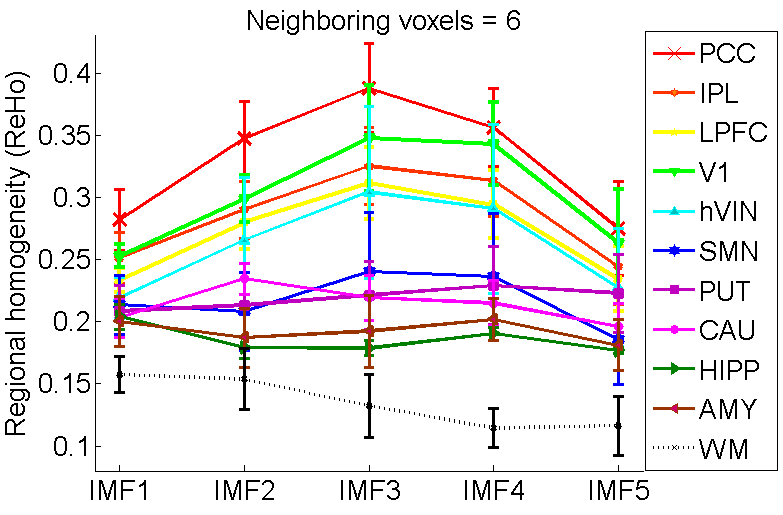

Supplement: Figure S1 — Frequency-specific ReHo in different brain regions, ReHo calculated with cluster size k = 7. (TIF) [file pone.0086818.s001.tif]

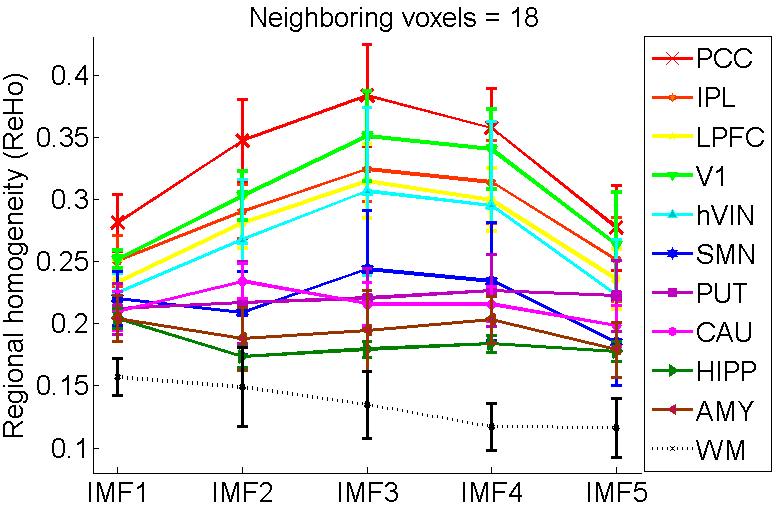

Supplement: Figure S2 — Frequency-specific ReHo in different brain regions, ReHo calculated with cluster size k = 19. (TIF) [file pone.0086818.s002.tif]
